# Meta - and combined - QTL analysis of different experiments on immune traits in chickens

**DOI:** 10.1007/s13353-013-0177-6

**Published:** 2013-10-11

**Authors:** Anna Slawinska, Maria Siwek

**Affiliations:** Department of Animal Biotechnology and Histology, University of Technology and Life Sciences, Mazowiecka 28, 85-084 Bydgoszcz, Poland

**Keywords:** Chicken, Combined, Immune response, Meta QTL analysis

## Abstract

Meta and/or combined QTL analysis from multiple studies can improve quantitative trait loci (QTL) position estimates compared to the individual experiments. Hereby we present results of a meta-analysis of QTL on chicken chromosome 9, 14 and 18 using data from three separate experiments and joint QTL analysis for chromosome 14 and 18. Meta QTL analysis uses information from multiple QTLs studies. Joint QTL analysis is based on combining raw data from different QTL experimental populations. QTLs under the study were related to specific antibody response to keyhole lymphet hemocyanin (KLH), and natural antibodies to environmental antigens, lipopolisaccharide (LPS) and lipoteichoic acid (LTA). Meta QTL analysis resulted in narrowing down the confidence interval for two QTLs on GGA14. The first one for natural antibodies against LTA and the second one for specific antibody response toward KLH. Also, a confidence interval of a QTL for natural antibodies against LPS located on GGA18 was narrowed down. Combined QTL analysis was successful for two QTLs: for specific antibody response toward KLH on GGA14, and for natural antibodies against LPS on GGA18. The greatest statistical power for QTL detection in joint analysis was achieved when raw data from segregating half–sib families from different populations under the study was used.

## Introduction

The major goal of quantitative trait locus (QTL) analysis is to describe the genetic basis for a trait of interest. The classical approach is based on whole genome scan, which is performed on an informative resource population established for the purpose to map QTL. The power of QTL detection is usually determined by the size of the experimental population. Confidence intervals of such QTL detected with whole genome scan approach are usually very large. The best approach to narrow down a QTL confidence interval is fine mapping. However, this method demands additional markers, new genotyping and new statistical analysis. There are two methods to improve QTL estimate using already available information. The first option is meta (QTL) analysis, second is a combined (QTL) analysis. The first alternative, meta QTL analysis, is based on a bibliographic survey and integrating information from multiple quantitative trait loci (QTLs) studies. Pooling of results from several studies allows greater statistical power for QTL detection and more precise estimation of their genetic effects (Wu and Hu 2012). Meta QTL analysis approach has been mostly used for plant (Shinozuka et al. 2012; Yadava et al. 2012; Sun et al. 2012) but also for pig data (Silva et al. 2011). The other alternative, joint QTL analysis, is based on combining individuals from several experimental populations, and increasing the number of individuals and the size of resource population. This approach has already been successfully used for joint QTL analysis in pigs (Rückert and Bennewitz 2010; Walling et al. 2000) and dairy cattle (Bennewitz et al. 2003). Bennewitz et al. (2003), has shown that combined QTL analysis increases statistical power due to increased half–sib family size. The goal of this study was to investigate the possibility to increase statistical power of QTLs and narrow down QTL confidence intervals by applying two statistical approaches: meta QTL and combined QTL analysis to QTLs for immune traits on three chicken chromosomes: GGA9, GGA14 and GGA18 from three different experiments.

## Material and methods

### Experimental populations

In meta and joint QTL analysis, data was used from three experimental populations; HiLo: chicken lines selected for high and low antibody response towards SRBC (as described by Siwek et al. 2003a); FP: selected for behavioral traits towards feather pecking (as described by Buitenhuis et al. 2003) and WLZk: a cross between commercial White Leghorn and native polish breed Green-legged Partridgelike (as described by: Siwek et al. 2010). All of the populations were created by inter se mating of F_1_ individuals. The number of half-sib families was six (HiLo, WLZk) or seven for FP population. The size of the F_2_ generation in these populations was as follows: HiLo: 672, FP: 630 and WLZk: 506 individuals.

### Phenotypic traits

Immune responses were defined as specific antibody response to keyhole lymphet heamocyanin (SAb-KLH), and natural antibodies (NAb) to environmental antigens lipopolisaccharide (LPS) and lipoteichoic acid (LTA). Phenotypic data was expressed by titers as the log_2_ values of the highest dilution giving a positive reaction as described by Siwek et al. 2003b (for KLH) and by Siwek et al. 2006 (for LTA and LPS). Phenotypic data was analyzed by the same method and laboratory for all three populations.

### Microsatellite markers

Initial QTLs under the study were located on GGA9 (NAb LPS), GGA14 (SAb-KLH, NAb LTA) and GGA18 (NAb LPS). Positions of the initial QTLs in each experimental population are presented in Table 1. All experimental populations were genotyped with the same microsatellite markers distributed over three chicken chromosomes: GGA9 (six markers), GGA14 (seven markers for WLZk population, four markers for HiLo/FP population), GGA18 (four markers).

**Table 1:** Description of QTLs subjected to meta and combined QTL analysis. CI – confidence interval of QTL region. LC – line cross model. HS – half –sib model with sire or dam common parent. LPS – natural antibodies against lipopolisaccharide. LTA – natural antibodies against lipoteichoic acid. SAb- KLH primary antibody response towards keyhole lymphet heamocynanin. HiLo – F_2_ experimental chicken population selected for primary antibody response towards sheep red blood cells. FP - F_2_ experimental chicken population selected for a feather pecking behavioral trait . WLZk F_2_ experimental chicken population obtained by crossing White Leghorn and Green legged Partridgelike. In bold, QTLs which were used in meta QTL analysis only

Chromosome	Marker	cM	Marker	cM	CI	Model	Trait	Population
**9**	**ROS0078**	**0**	**GTC0016**	**41**	**0–41**	**LC**	**LPS**	**HiLo** ^*^
**9**	**ROS0078**	**0**	**GTC0016**	**41**	**0–41**	**LC**	**LPS**	**WLZk ** ^*^
14	MCW0123	45	MCW0225	77	45–71	HS sire	SAb - KLH	WLZk^#^
14	MCW0123	45	MCW0225	77	45–77	HS/LC	SAb – KLH	HiLo^**^
14	MCW0136	20	MCW0123	45	0–45	HS dam	SAb - KLH	WLZk^*^
**14**	**MCW0296**	**0**	**MCW0136**	**20**	**0–20**	**LC**	**LTA**	**FP** ^*^
**14**	**MCW0296**	**0**	**ADL0118**	**20**	**0–20**	**HS sire**	**LTA**	**FP** ^*^
**14**	**MCW0123**	**45**	**MCW0225**	**77**	**45–77**	**LC**	**LTA**	**HiLo** ^*^
18	MCW0045	0	ROS0022	24	0–24	HS sire	LPS	FP^*^
18	MCW0045	0	MCW0217	25	0–25	HS sire	LPS	WLZk^*^

^**^-QTL statistically significant at *P* < 0.01

^*^-QTL statistically significant at *P* < 0.05

^#^-QTL non-significant

### Statistical analysis - software

Meta QTL analysis was done based on the literature information using Metaqtl software (Veyrieras et al. 2007 (available on http://www.bioinformatics.org/mqtl)) according to protocol described by Silva et al. 2011. Combined/joint QTL analysis was performed with a half-sib model implemented in GridQTL (Seaton et al. 2006) software where the data from half-sib families of all three experimental populations was used. Combined QTL analysis concerned two by two experimental populations: HiLo and WLZk for QTL on GGA14, and FP and WLZk for QTL on GGA18. Joint QTL was performed in two steps: at first data from all half sib families of two experimental populations was used, secondly: only segregating half sib families entered the QTL analysis.

## Results

Chicken QTL data base (http://www.animalgenome.org/cgi-bin/QTLdb/GG/index) contains 413 QTL related to disease susceptibility. However, our traits of interest: primary antibody response toward KLH, natural antibodies for LPS and LTA, were analyzed in three experimental populations only (FP, HiLo and WLZk).

### Meta QTL analysis

**Fig. 1:**
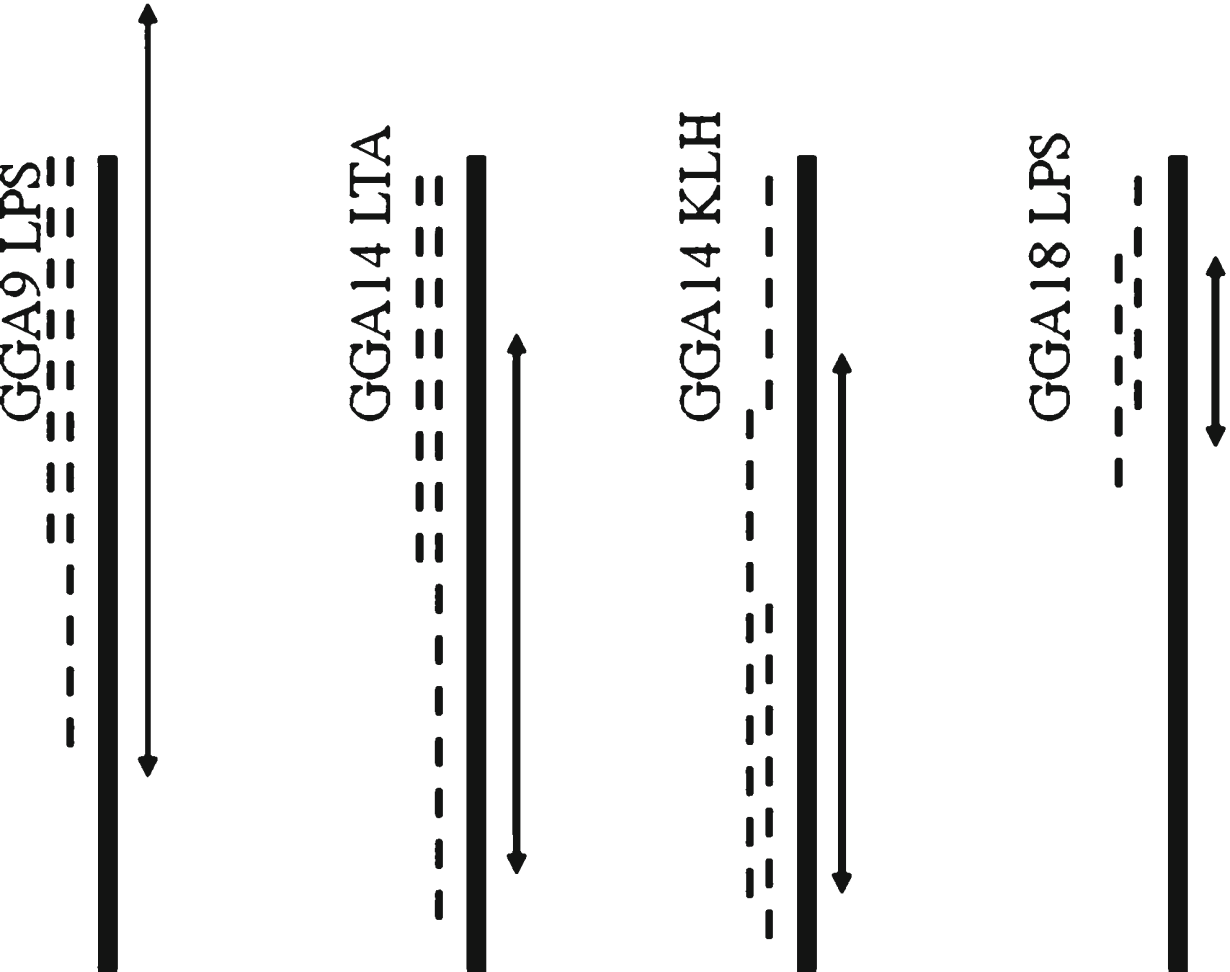
QTL confidence interval for meta QTL analysis on chicken chromosome GGA9, GGA14 and GGA18. LPS - indicates QTL associated with lipopolysaccharide natural antibodies; LTA – indicates lipoteichoic acid antibodies; KLH – indicates primary antibody response toward keyhole lymphet heamocyanin. Solid bar presents the length of each chromosome. Dotted line shows confidence interval of the original QTL located on this chromosome in one of the three reference populations (Green-legged Partridgelike and White Leghorn cross; feather pecking cross; population selected for primary antibody response towards SRBC). *Arrow* indicates new confidence interval defined in meta QTL analysis

Meta QTL analysis was performed for QTLs on three chicken chromosomes: GGA9, GGA14 and GGA18 (Fig. 1). Meta QTL for LPS natural antibodies on GGA9 is located in marker bracket: ROS0078 – GCT0016. Chicken chromosome 14 harbors two meta - QTLs: the first one associated with primary antibody response toward KLH and the second one related to LTA NAb. Both meta QTL are located between markers: ADL0200 and ROS0284. The second meta QTL associated with LPS natural antibodies is located on GGA18 in marker bracket: ADL0304 – ADL0290. All detected meta QTLs were located within confidence intervals of initial QTL entering the meta QTL analysis.

### Combined QTL analysis

**Fig. 2:**
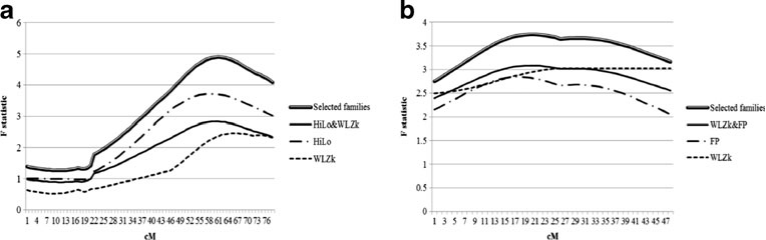
**a**. Test statistic for GGA14 with regard to the specific primary antibody response to keyhole lympet heamocyanin (KLH) under half sib analysis model with sire as common parent for the F2 crosses of two reference populations. The  line describes test statistic for Green-legged Partridgelike and White Leghorn population (WLZk). The  line describes test statistic for population selected for primary antibody response toward SRBC (HiLo). Solid lines shows test statistic for combined QTL analysis of WLZk and HiLo populations. *Double line* presents test statistic for combined QTL analysis of segregating families from two reference populations (WLZk and HiLo). The X axis is a length of the chromosome in cM. **b**. Test statistic for GGA18 with regard to lipopolisaccharide natural antibodies (LPS) under half sib analysis model with sire as common parent for the F2 crosses of two reference populations. The  line describes test statistic for Green-legged Partridgelike and White Leghorn population (WLZk). The  line describes test statistic for feather pecking population (FP). Solid lines shows test statistic for combined QTL analysis of WLZk and FP populations. *Double line* presents test statistic for combined QTL analysis of segregating families from two reference populations (WLZk and FP). The X axis is a length of the chromosome in cM

Combined QTL analyses were performed for two QTLs: one QTL on GGA14 (Fig. 2a) and the second one on GGA18 (Fig. 2b). Both figures present four curves. First and second curve represents a test statistic for initial QTLs detected in each experimental population segregating QTL alleles. Third profile is a test statistic for combined QTL analysis using all half–sib families from two experimental populations HiLo & WLZk on GGA14 and FP & WLZk on GGA18. Finally, the last, top graph is a test statistic of combined QTL analysis for families segregating QTL alleles in two experimental populations. Combined QTL analysis performed on data from two experimental (WLZk, HiLo) populations on GGA14 allowed to increase statistical power of QTL for SAb-KLH. Initial F values were as follows: 2.46 for WLZk population (QTL statistically non-significant); and F value = 3.72, for HiLo population (statistically significant at P < 0.01). Combined QTL analysis of five half-sib families from WLZk population and 2 half-sib families from HiLo population gave statistically significant QTL at *P* < 0.01 with F value = 4.90.

The second combined QTL analysis has been performed for a QTL for LPS NAbs located on GGA18. Joint QTL analysis of raw data of segregating half-sib families from WLZk population (four half-sib families) and FP population (3 half-sib families) allowed to increase the F value to 3.74 and statistical significance to *P* < 0.01. Initial QTLs located on GGA18 have the statistical significance level at *P* < 0.05 and F values equal 2.84 for FP population and 3.02 for WLZk population. Combined analysis of all half-sib families from two populations improved statistical significance of detected QTL to *P* < 0.01 and the F value equal to 3.09.

## Discussion

QTLs detected for SAb-KLH (Siwek et al. 2003a), NAb LPS and NAb LTA (Siwek et al. 2006) in two experimental populations, HiLo and FP, were validated in the third resource population the WLZk cross (Siwek et al. 2010; Slawinska et al. 2011). In the current study we went one step forward and performed a meta QTL and joint QTL analysis based on observations and raw data respectively. The ultimate goal of the QTL studies is finding the causative mutation underlying change in the phenotype. However, quantitative traits, such as immune response, are controlled by many genes along with the environment (Dekkers 2012). Therefore, deciphering the gene or genes related to a change in phenotype is a great challenge. QTL confidence intervals are usually quite large and localization of QTL peak is not very precise. All of these make the positional and biological candidate gene analysis more difficult. As it has been already mentioned, a WLZk experimental population was used to validate QTLs related to immune responses toward KLH, LTA and LPS. The same population is also subjected to SNP genotyping and the associated study of the biological candidate genes. For this reason, narrowing QTL region and defining QTL peak position with good precision was of primary interest. Two statistical approaches, meta and combined QTL analysis, allowed to increase the significance of a QTL (for two QTLs), narrow down QTL confidence interval and improve precision of QTL peak position (for three QTLs). Efficiency of meta analysis depends on number of studies used and their heterogeneity (Berman and Parker 2002). In our case, the number of studies was limited and their heterogeneity was rather low. Except from different experimental populations used in each QTL study, the remaining factors were the same: microsatellite markers, phenotyping procedure; or very similar: trait heritability, QTL effect. Similar meta QTL results on limited number of initial QTL studies were obtained by Silva et al. 2011. These authors analyzed QTLs for meat quality traits on pig chromosome 4. In the case of two initial QTLs for trait growth, calculated meta QTL had large interval (Silva et al. 2011).

Meta QTL on chicken chromosome 9 has a very particular feature. The meta confidence interval exceeds the length of the chromosome and is longer than two initial confidence intervals, which is biological impossible (Fig. 1). The reason for this is location of the initial QTL peak at 0 cM, at the same position as its flanking marker. The Metaqtl software considers this flanking marker as missing, and therefore creates an extended meta confidence interval (Veyrieras et al. 2005). It might also mean, that meta QTL analysis using Metaqtl software might be challenging for QTLs located at the distal part of the chromosomes. It should also be keep in mind that meta QTL analysis is based on published, therefore, positive results only. QTL data which enters meta analysis are all significant. As has been presented by Silva et al. 2011 in Tables 5 and 6, meta QTL analysis narrows down the confidence interval of the chromosomal region linked with the trait of interest, and decreases the number of meta-QTLs for each trait compared to number of trait QTLs detected in each independent population. Nevertheless, these results might be biased by the lack of negative QTL results entering the meta QTL analysis.

Combined QTL analysis has been performed in two steps. First step involved raw data of all families from two experimental populations. This combined QTL analysis has proven a QTL location chromosomal location in both populations. Second step could be seen as a fine mapping QTL analysis. In this step only segregating half-sib families were subjected to the combined QTL analysis. In this case seven half-sib families from two experimental populations were used. The number of half-sib families selected for joint QTL analysis is in agreement with the number of families suggested by Wu and Jannik (2004). Based on the simulation studies conducted by these authors, the greatest power of QTL mapping was found when five to ten families are used. In the case of reported study, using segregating families from two experimental populations in combined QTL analysis resulted in significantly higher F value of detected QTL. However, the obtained confidence interval has not decreased. The most likely reason is low number of microsatellite markers genotyped for each of the experimental populations.

Joint QTL analysis has already been performed for pigs (Rückert and Bennewitz 2010; Walling et al. 2000). Rückert and Bennewitz in their joint QTL analysis, used a very specific set up where founder breeds of several F_2_ crosses are the same. However, the second study by Walling et al. (2000) is using independent F_2_ crosses of various pig breeds. Both studies demonstrate great potential of joint QTL analysis in increasing significance of detected QTLs. Walling et al. (2000) suggested in their manuscript that ideally all populations used for joint QTL analysis would have measured the same traits and genotyped for the same markers. Our combined QTL analysis met these demands.

As has been mentioned already, the experimental populations share many features. Another common characteristic is their layer origin. It has been shown by Megens et al. (2009) that layers have large linkage disequilibrium blocks, which justifies using this resource population for finding association between markers and phenotypes.

A study by Olkin and Sampson (1998) showed that meta-analysis of summary statistics can be as powerful as analysis of the combined data. Presented results seem to confirm this statement. However, some limitations should be considered. Meta QTL analysis works better for a large number of initial studies with large heterogeneity. Joint QTL analysis, if using half–sib, model demands initial QTL to be detected under the same QTL model.

To the authors’ knowledge, the presented study is the first meta and joint QTL analysis of immune related traits in chicken. The outcome of meta and joint QTL analysis has already been an indication for candidate gene selection on three chicken chromosomes. Potential association of these genes with immune traits of interests will be subsequently validated based on SNP information in WLZk reference population.
